# A Review of Economic Evaluations of Tobacco Control Programs

**DOI:** 10.3390/ijerph6010051

**Published:** 2008-12-28

**Authors:** Jennifer W. Kahende, Brett R. Loomis, Bishwa Adhikari, LaTisha Marshall

**Affiliations:** 1 Centers for Disease Control and Prevention, National Center for Chronic Disease Prevention and Health Promotion, Office on Smoking and Health, 4770 Buford Highway, NE., MS-K50, Atlanta, GA 30341, USA; E-mails: Badhikari@cdc.gov (B. A.); Lmarshall@cdc.gov (L. T. M.); 2 Research Triangle International, Public Health Policy Research Program, Hobbs Building, Rm 139, 3040 Cornwallis Rd, Research Triangle Park, NC 27709, USA; E-Mail: Loomis@rti.org

**Keywords:** Economic evaluation, cost-effectiveness, smoking, tobacco use, smoking cessation

## Abstract

Each year, an estimated 443,000 people die of smoking-related diseases in the United States. Cigarette smoking results in more than $193 billion in medical costs and productivity losses annually. In an effort to reduce this burden, many states, the federal government, and several national organizations fund tobacco control programs and policies. For this report we reviewed existing literature on economic evaluations of tobacco control interventions. We found that smoking cessation therapies, including nicotine replacement therapy (NRT) and self-help are most commonly studied. There are far fewer studies on other important interventions, such as price and tax increases, media campaigns, smoke free air laws and workplace smoking interventions, quitlines, youth access enforcement, school-based programs, and community-based programs. Although there are obvious gaps in the literature, the existing studies show in almost every case that tobacco control programs and policies are either cost-saving or highly cost-effective.

## 1. Introduction

Cigarette smoking imposes an enormous health and economic burden on the nation. Each year, an estimated 443,000 people die of a smoking-related disease in the United States [1]. Smoking is the responsible for at least 80% of deaths from chronic obstructive pulmonary disease (COPD) and early cardiovascular disease and death, and 30% of all cancer deaths [2]. Cigarette smoking results in more than $193 billion in medical costs and productivity losses annually [1]. In 2001, states spent $12 billion in Medicaid program funds to treat tobacco-related diseases [3]. The total costs of cigarette smoking to the Medicare program amounted to $20.5 billion in 1997 [4]. In an effort to reduce this burden, many states, the federal government, and several national organizations fund tobacco use prevention and control programs. The Centers for Disease Control and Prevention (CDC) recommends an average of $5.98 per person in tobacco control program spending for states [5]. In fiscal year 2006, states spent an estimated $538 million ($1.85 per person) on tobacco control programs, down from a peak of $750 million ($2.51 per person) in fiscal year 2003 [6]. Tobacco control programs and policies are effective at reducing smoking initiation, increasing smoking cessation, and reducing exposure to secondhand smoke [7–8]. Although much is understood about the effectiveness of tobacco control policies, comparatively little is known about their cost-effectiveness. Economic evaluation is useful in identifying, measuring, valuing and comparing the costs and consequences of alternative interventions being considered. For this report we reviewed existing literature on economic evaluations of tobacco control interventions. Although there are obvious gaps in the literature, the existing studies show in almost every case that tobacco control programs and policies are either cost-saving or highly cost-effective when compared with other public health interventions. We anticipate that this report will assist researchers, tobacco control advocates and decision makers in setting an economic research agenda that makes the case for sustaining state tobacco control programs.

## 2. Methods

We reviewed 42 papers from the peer-reviewed literature on economic evaluations of tobacco control programs and policies. We captured economic evaluation studies published between 1983 and 2006 by using searches on PubMed, EconLit, ScienceDirect, and JSTOR. Cost per quitter, cost per life year saved, and cost per quality adjusted life year were extracted from articles that reported those outcomes. Studies that reported other outcomes were also summarized. We excluded papers that describe only the effectiveness of an intervention, without consideration of costs. Of the 42 studies we summarized, 26 are cessation interventions. By far, smoking cessation therapies, including nicotine replacement therapy (NRT) and self-help are most commonly studied. There are far fewer studies on other important interventions, such as price and tax increases (4), media campaigns (2), smoke free air laws and workplace smoking interventions (2), quitlines (2), youth access enforcement (1), school-based programs (2), and community-based programs (3). Cost estimates are expressed in 2005 US dollars by adjusting for inflation. Figures originally reported in British pounds were first converted to US dollars using the prevailing conversion rate at the time the cost data were collected, and then were adjusted for inflation.

## 3. Elements of Economic Evaluation

We now briefly describe the four main methods used in economic evaluations: cost analysis (CA), cost-effectiveness analysis (CEA), cost-utility analysis (CUA), and cost-benefit analysis (CBA).

### 3.1. Cost Analysis

Cost analyses can be conducted alone, but they are often part of CEA, CBA or CUA and include the costs of developing and implementing an intervention. Costs are typically expressed as total dollars or dollars per person served by the program [9]. These costs are classified as direct costs, indirect costs, and intangible costs. Direct costs can be either medical or nonmedical. Indirect costs (opportunity costs) are related to the time and lost productivity of those who are targeted by the intervention [10]. Intangible costs, which measure the pain or suffering associated with an intervention, are difficult to quantify and therefore are seldom included in economic evaluations [10].

### 3.2. Cost-Effectiveness Analysis

CEA links the costs of an intervention to the health improvements caused by the intervention [8]. Measures of health improvement include cases avoided, hospital days avoided, deaths averted, and life years saved. CEA is used to compare a single intervention with no intervention or to compare two or more interventions with differing levels of effectiveness or cost [11]. The health improvements resulting from each intervention do not need to be the same, but they must be suitable for conversion to a common unit, such as life years saved. Results from a CEA are typically expressed as a cost-effectiveness ratio, which measures the net cost of the intervention per unit of health gained. There are three types of cost-effectiveness ratios: the incremental cost-effectiveness ratio (ICER), which measures the cost to gain an additional unit of health from one intervention versus another intervention. Marginal cost effectiveness ratio (MCER) measures additional outcomes from additional investments in an intervention, and the average cost-effectiveness ratio (ACER) which compares a given intervention to no intervention [10].

### 3.3. Cost-Utility Analysis

CUA compares costs of an intervention with one particular measure of health improvement, the quality adjusted life year (QALY). QALY takes into account both mortality and morbidity to assess the overall quality of life. Results of CUAs are typically expressed as cost per QALY saved [9]. The advantage of this approach is that it allows different types of health improvements to be compared [10]. Two other time-based measures of health often associated with CUA are the disability-adjusted life year (DALY) and the healthy life year (HeaLY).

### 3.4. Cost-Benefit Analysis

CBA expresses both the costs of the program and the health gains achieved in dollars, which are adjusted to their current or present value through discounting. Discounting is a process that makes the value of costs and benefits comparable regardless of when they occur [12]. CBA is used when the interventions being compared result in differing or multiple outcomes. The two most commonly used summary measures for CBA are net benefits (present value of benefits less harms, minus cost of prevention) and benefit–cost ratio (present value of benefits divided by present value of costs) [10]. Difference in interest rates assumed will have great impact on present value of future stream of benefits. In general, if the benefits exceed the cost, investment in the program is economically sound. CBA is not as common in public health research as CEA and CUA because of the difficulties and controversy that can surround putting monetary values on health outcomes.

### 3.5. Role of Perspective in Economic Evaluation

Each of the economic evaluation studies described above (CA, CEA, CUA, and CBA) can be carried out from the perspective of different stakeholders in the intervention, such as intervention participants, the funding agency, or society as a whole [10–11]. The perspective of an economic evaluation is often determined by the end user of the study, and perspective in turn determines which costs and benefits should be included in the study. For this reason, it is critical to specify the perspective of the study before beginning data collection or analysis. Common perspectives used include private payers, government agencies, public payers, or society as a whole. Decision makers with a fixed budget, a set of options for using the budget, and a series of other constraints (resource, ethical, or political) often want evaluations that are highly specific to their context [9–11].

## 4. Results

### 4.1. Policy Interventions

#### 4.1.1. Cigarette tax and price increase

The costs of a cigarette tax (or price) increase are essentially zero from the public health perspective. However, gains to society from an increase in cigarette tax revenue and health care dollar savings offset the potential costs borne by smokers, retailers, and producers. Although smuggling and tax avoidance are serious issues, every cigarette tax increase has historically resulted in an increase in revenue for the government. We found three studies that used simulation models to estimate the impact of a cigarette price or tax increase on health and economic outcomes [13, 14, 16] Ahmad [13] developed a simulation model for the population of California over a 75-year period and found that with a 20% cigarette tax increase a reduction in smoking prevalence from the current level of 17% down to 12% could be projected. Over 75 years, tax revenues would increase by almost $12 billion, and smoking-attributable medical costs would decrease by $220 billion. Fishman *et al.* [14] estimated the cost-effectiveness of the combined effects of a national mass media antismoking campaign and a $1 increase in the federal cigarette tax using the model published by Rivara *et al.* [15]. They found that the number of life years saved by the tax increase and media campaign was 630,925. Accounting only for the costs of the media campaign, the cost per life year was between $599 and $4,646. Factoring in the increase in revenue from the $1 tax increase makes the combined intervention cost saving of $573,346 to $797,701 per life year saved.

Kaplan *et al.* [16] modeled changes in smoking prevalence in response to a range of cigarette tax increases in California one year after the tax and 75 years into the future. Their model was static and did not simulate change in the population over time. Rather, the authors assumed that the effects of a cigarette tax increase today on the smoking population 75 years from now would be entirely through a reduction in smoking initiation. Assuming an elasticity of cigarette demand of –0.4, a $0.50 tax increase would lead to a purchase of 8,389 QALYs in the first year. Expected benefits were higher until a steady level was attained after 75 years (52,136 QALYS per year). A $1 tax increase and a -0.60 elasticity would result in 25,380 QALYS saved in the first year. A greater (smaller) elasticity of demand would result in more (fewer) QALYs saved.

We also identified an international study that used traditional cost-effectiveness techniques to evaluate a cigarette price increase [17]. Those investigators calculated the cost-effectiveness of price increases, NRT, and a package of non-price interventions other than NRT (e.g., advertising bans, smoke-free-air laws, media campaigns). The authors found that a 10% tax-induced increase in cigarette prices worldwide could prevent between five and 16 million tobacco-attributable deaths. They also found price increases in high income countries had a cost-effectiveness of $106 – $3,550 per DALY, NRT $961 – $9,231 per DALY, and other non-price interventions $892 – $17,837 per DALY.

All these studies show that cigarette tax increases are unique in that they are highly effective at reducing the health and economic burden of smoking while at the same time generating higher revenue for the government.

#### 4.1.2. Smoke-free-air laws and policies

A systematic search of the existing literature was unable to identify any studies or reviews pertaining specifically to the cost-effectiveness of smoke-free-air laws. The Community Guide cited only one cost-benefit study on smoke-free-air laws [18]. This study is a non-peer-reviewed governmental report that models the benefits and costs of a proposed national smoke-free environment act to restrict or ban smoking in all public buildings regularly entered by ten or more people per week. Costs included implementation of the restriction, construction and maintenance of smoking lounges, and enforcement. Benefits included savings on medical expenditures by averting heart disease, the value of lives saved, costs averted by reduced smoking-related fires, and productivity improvements. The net present benefit to society was between $42 and $78 billion, and this range was based on high and low estimates of costs and benefits. Ong and Glantz [19] compared the cost-effectiveness of NRT with a statewide smoke-free workplace policy in Minnesota. The study found that an NRT program would generate 18,500 quitters at a cost of $10,067 per quitter ($6,367 per QALY), whereas a smoke-free workplace policy would generate 10,400 quitters at a cost of $1,146 per quitter ($726 per QALY). They concluded that a statewide workplace smoking policy would be about nine times more cost-effective than a statewide NRT program. There is extensive literature assessing the economic impact of smoking bans and restrictions. For example, Mandel *et al.* [20] concluded that smoke-free laws are associated with no change in gaming revenue in Delaware. Scollo *et al.* [21] reported that smoke-free restaurant and bar laws had no impact on sales and employment in their review of all studies pertaining to the impact of smoke-free-air policies on the hospitality industry. The Surgeon General’s report on the health consequences of involuntary exposure to tobacco smoke includes evidence from studies showing smoke-free policies have no effect on revenue and in some cases there have been increases in revenue [22–29]. There is evidence of changing attitudes among bar patrons and owners and staff in favor of smoke-free-air policies and high rates of compliance [30–32]. Given the success of smoke-free-air policies, an important next step and a current omission from existing literature is an evaluation of the cost-effectiveness of these measures.

#### 4.1.3. Insurance coverage

We found only one study on cost-effectiveness of workplace smoking interventions. In that study Curry *et al.* [33] compared the cost-effectiveness of four employers’ health care coverage plans and concluded that as many as 2.8% of employees would quit if both behavioral cessation methods and nicotine replacement methods were fully covered. Offering full coverage would increase the cost to employers from $2.10 to $6.48 per enrollee per year. Average total cost per smoker who quit ranged from $1,223 to $1,571.

#### 4.1.4. Youth access enforcement

DiFranza *et al.* [34] modeled the vigorous enforcement of laws banning the sale of tobacco to minors in every state. In the absence of good efficacy data on youth access enforcement, they asked, “How effective at reducing the prevalence of youth smoking would youth access enforcement have to be in order to make it cost-effective?” If the enforcement program resulted in a 5% reduction in the prevalence of youth smoking, the cost per life year saved would range from $535 to $3,773, depending on the costs of enforcement. If the enforcement program resulted in a 25% reduction in the prevalence of youth smoking, the cost per life year saved would range from $107 to $742.

### 4.2. Population-Based Interventions

#### 4.2.1. Mass media campaigns

Two studies were identified that examined the cost-effectiveness of mass media campaigns. Ratcliffe, Cairns, and Platt [35] evaluated the costs and outcomes of Scotland’s general public antismoking campaign. The campaign consisted of three main components: (1) mass media advertising, including television, outdoor posters, and press; (2) *Smokeline*, a telephone quitline for cessation support; and (3) *You can stop smoking,* a booklet providing practical advice on giving up smoking. The cost per quitter ranged from $341 to $748. The cost per life year saved ranged from $617 to $1,330 at a 6% discount rate. Secker-Walker *et al.* [36] estimated the cost-effectiveness of a four-year mass media campaign that was shown to prevent the onset of smoking in adolescents. The media campaign was added to a school-based tobacco education curriculum. The media campaign was aired for four years, beginning in grades 5 through 7 (ages 10 to 13) and continuing through grades 8 through 10 (ages 13 to 16). The cost of developing the campaign was $945,498; and the cost per student potentially exposed (to the campaign (*n* = 18,600) was $51. The cost per smoker averted (*n* = 1,023) was $939, and the cost per life year saved discounted at 3% was $867.

#### 4.2.2. Community-based interventions

Secker-Walker *et al.* [37] estimated the cost-effectiveness of a four-year, multifaceted, community-based research project designed to help women aged 18 to 64 quit smoking (the *Breathe Easy* project). The study employed a quasi-experimental matched control design and compared women living in two counties in Vermont and two counties in New Hampshire. The cost-effectiveness of per life year saved, discounted at 5%, was $2,087 (90% CI: $1,112, $16,993) for the intervention. Tran *et al.* [38] compared the cost-effectiveness of a smoking cessation program in a community pharmacy practice with the cost-effectiveness of a self-directed quit attempt. The incremental cost for an additional patient to quit smoking using the pharmacist-directed program alternatives, compared with a self-directed quit attempt, was $277 for the self without NRT method, $1,097 for the nicotine patch, $2,109 for nicotine gum, and $1,348 for bupropion. Depending on the smoker’s age at the time of cessation, the incremental discounted cost-effectiveness was $844 to $1,662 per life year saved. This analysis suggests that a pharmacist-directed cessation program is a cost-effective alternative to a self-directed quit attempt.

Crealey *et al.* [39] studied the cost-effectiveness of a community pharmacy-based smoking cessation program employing data from a pilot study in two community pharmacies in Northern Ireland. The program was a four-stage process that included a written contract between pharmacist and patient with a defined “stop date”, with a series of counseling meetings over a period of six months. The authors found that the cost per life year saved ranged from $393 to $701 for men and from $362 to $1,541 for women, depending on age. This result compared favorably with other disease prevention medical interventions, such as screening for hypertension or hypercholesterolemia.

#### 4.2.3. Quitlines

We found two studies that evaluate the cost-effectiveness of quitlines. McAlister *et al.* [40] used a randomized trial to evaluate the American Cancer Society’s telephone counseling service to assist smokers willing to quit. The study found that counseling nearly doubled a smoker’s odds of quitting and maintaining cessation status for one year. They concluded that the cost for each case of maintained cessation attributable to counseling availability was approximately $1,475 yearly. Tomson *et al.* [41] evaluated the cost-effectiveness of the free Swedish nationwide quitline service. The study included 1,131 callers enrolled in the program from 1 February 2000 to 30 November 2001. The authors found that the cost per quitter was $1,161 **–** 1,500 and $343 **–** 443 per life year saved. They concluded that the national quitline program was cost-effective.

### 4.3. Individual Interventions

#### 4.3.1. School-based tobacco use prevention programs

Two studies were identified that examined the cost-effectiveness of school-based antitobacco education programs. Tengs, Osgood, and Chen [42] modeled the cost-effectiveness of an intensive school-based tobacco education implemented nationwide using the Behavioral Risk Factor Surveillance System (BRFSS) survey data and data on fertility, mortality, health-related quality of life, cost of educational programs, and the cost of medical care. They concluded that the cost-effectiveness of a school-based tobacco education program would be $23,440 per QALY purchased if this program had 30% effectiveness that dissipated in four years. Wang *et al.* [43] estimated the cost-effectiveness of the Project Toward No Tobacco Use (TNT) intervention. TNT consists of a ten-lesson curriculum delivered by trained health educators to a cohort of 1,234 7^th^ grade students in eight junior high schools. At a total cost of $24,506, the TNT intervention prevented 35 students from becoming established smokers. This effect resulted in an estimated cost savings of $19,894 per life year purchased and $12,672 per QALY purchased.

#### 4.3.2. Nicotine replacement therapy and bupropion

We reviewed seven studies that examined the economic evaluations of NRT and bupropion, which are often combined with counseling [44–50]. Croghan *et al.* [44] estimated the cost-effectiveness of nicotine dependence treatment, including NRT and counseling. The overall one-year smoking cessation rate was 22.2% with a cost of $9,231 per net life year gained. Compared with other medical services, these interventions are relatively inexpensive and cost-effective. Friscella and Franks [45] investigated the incremental cost-effectiveness of the transdermal nicotine patch with physician advice versus physician advice alone. They found that use of the patch produced one additional lifetime quitter at a cost of $9,392. The incremental cost-effectiveness of the patch ranged from $5,624 to $14,018 per QALY for men and from $6,347 to $8,945 for women. They found that a clinical strategy that limited prescription renewals to patients who had successfully abstained for two weeks improved the cost-effectiveness of the intervention by 25%. Gilbert *et al.* [46] examined the incremental cost-effectiveness of pharmacological smoking cessation therapies. They found that the cost per life year saved was $4,031 for nicotine gum, $2,152 for the nicotine patch, $4,992 for nicotine spray, $4,660 for the nicotine inhaler, and $1,438 for bupropion. The authors concluded that pharmacological therapies could be cost-effective as compared with other medical interventions.

Hughes *et al.* [47] monitored 106 smokers who received physician counseling and were randomized to receive a prescription for nicotine gum at varying levels of cost ($30, $9, and $0). Lower cost for nicotine gum was associated with higher incidence of obtaining nicotine gum, increased cessation attempts, and increased abstinence after six months. CBA suggests a net benefit of $1,673 per quitter for the free gum, $418 per quitter for the $9 gum, and $617 per quitter for the $30 gum. Stapleton, Lowin, and Russell [48] calculated the incremental costs per life year saved of using the patches in addition to physician counseling. The incremental cost per life year saved would be $791 for individuals younger than 35 years, $686 for individuals aged 35 to 44 years, $859 for individuals aged 45 to 54 years, and $1,561 for individuals aged 55 to 65 years. Wasley *et al.* [49] used a randomized controlled trial design to determine the cost-effectiveness of the nicotine transdermal patch. The study found that using the patch in conjunction with counseling was more cost-effective than counseling alone. Depending on age, the incremental costs per life year saved when using the patch ranged from $2,301 to $3,778 for men and from $3,894 to $5,625 for women. Woolacott *et al.* [50] developed a decision analytic model to compare the cost-effectiveness of counseling only, counseling plus NRT, counseling plus bupropion, and counseling plus NRT and bupropion. The incremental cost per life year saved, as compared with advice only, was $2,540 to $7,621 for NRT, $1,580 to $4,743 for bupropion, and $2,085 to $6,256 for NRT and bupropion. In all seven studies, NRT proved to be highly cost-effective with an incremental cost per life year gained between $1,447 and $11,374.

#### 4.3.3. Counseling and group therapy

We reviewed four studies that focused on counseling, including group therapy, for smoking cessation. Some of the interventions included in these studies also used NRT. Each reviewed study provided evidence that counseling was a cost-effective treatment for smoking cessation, but they also suggested that including NRT might be more cost-effective than counseling alone.

Cromwell *et al.* [51] studied the cost-effectiveness of the 1996 Smoking Cessation Clinical Practice Guidelines [52]. They studied various forms of counseling with and without the nicotine patch and nicotine gum alone. Implementing the entire package of guidelines would cost almost $8.1 billion for the first year, producing 1.7 million quitters at an average cost of $4,841 per quitter. Cost per QALY ranged from $1,419 to $5,818. The most intensive interventions were the most cost-effective, suggesting that greater spending on cessation interventions produced more net benefit.

Cummings, Rubin, and Oster [53] analyzed the cost-effectiveness of physician counseling to quit during a routine office visit by using published reports from randomized trials. The authors found that brief counseling during routine office visits cost between $1,325 and $1,857 for men and between $2,264 and $3,869 for women per life year saved. The cost-effectiveness of counseling appeared to be equivalent to that of nicotine gum. The study concluded that counseling cessation was as cost-effective as many other preventive medical practices and should be incorporated into routine health care for patients who smoke. Krumholz *et al.* [54] evaluated the cost-effectiveness of a smoking cessation program delivered by nurses in the hospital to smokers who had been admitted for a myocardial infarction. They concluded that the incremental cost per life year saved of the program, compared with usual care, was approximately $306 per year.

Meenan *et al.* [55] evaluated the cost-effectiveness of a smoking cessation and smoking relapse prevention program for hospitalized adult smokers. This study was conducted from the perspective of the hospital. The authors used discounted costs per life year saved to measure the effectiveness of interventions which included a 20-minute bedside counseling session, a 12-minute video, self-help materials, and a number of follow-up calls. They found that the incremental cost per incremental quitter was $4,873 ($210 per smoker), with costs per life year saved ranging from $2,229 to $9,811. With routine implementation, total intervention costs would decrease dramatically and thus reduce costs per life year saved by as much as 90% to $501.

#### 4.3.4. Cessation interventions among pregnant women

The high costs of low birth weight (LBW) infants, premature deliveries, and sudden infant death syndrome (SIDS) deaths suggests that interventions to reduce smoking among pregnant women are likely to be highly cost-effective. We reviewed six economic evaluations [56–61] of cessation interventions among pregnant women, two of which estimated the break-even cost (the cost below which the intervention is cost-effective). These articles show that highly effective interventions could be implemented for as little as $16 to $50 per pregnancy, making them also highly cost-effective.

Ershoff *et al.* [56]compared home-based smoking cessation and nutrition program versus standard prenatal care and found women who received the smoking cessation program were more likely to quit during pregnancy (49.1% versus 37.5%), and those who did smoke throughout their entire pregnancy smoked fewer cigarettes per day. Women in the experimental group had infants of higher mean weight and had fewer LBW and preterm infants. The average cost per delivery for women in the experimental group was $1,001 as compared to $1,395 for women in the control group.

Ershoff *et al.* [57] studied cost-effectiveness in an experimental group of pregnant smokers who received education on the hazards of smoking while pregnant and a serialized cessation program versus pregnant smokers who were assigned to the usual care control group. On average, infants born to experimental group participants were 57 grams heavier, and the incidence of LBW was nearly halved in the experimental group as compared with the control group. The average cost per delivery was $1,767 for the experimental group as compared with $1,846 for the control group. Hueston, Mainous, and Farrell [58] examined the cost-effectiveness of smoking cessation programs during pregnancy to prevent LBW births, and a decision tree was constructed to estimate break-even costs, assuming a quit rate of 18%. To be cost-effective, a smoking cessation intervention had to decrease smoking rates by 2.15% to justify every $15.75 in intervention costs. Thus, for a program that resulted in 18% of smokers quitting when they otherwise would not, a cost of up to $126 per participant would be cost-effective.

Marks *et al.* [59] estimated the cost-effectiveness of a smoking cessation program for preventing LBW and prenatal mortality. The authors assumed that an effective program would cost about $53 per participant, 15% of whom were estimated to quit smoking. They estimated that the program would shift 5,876 LBW infants to normal birth weight at a cost of $7,128 per LBW infant. A comparison of costs of caring for LBW infants in a neonatal intensive care unit showed that smoking cessation programs would save $138,652,170 or $3.31 per $1 spent.

Pollack [60] analyzed the relationship between prenatal maternal smoking and SIDS and considered the cost-effectiveness of a hypothetical smoking cessation intervention that cost approximately $37 per participant and achieved a 15% cessation rate. The author found that 26% of singleton SIDS deaths were attributable to prenatal maternal smoking. Typical cessation services available to all pregnant smokers could avert 108 SIDS deaths per year at a cost of $262,073 per life saved. Shipp *et al.* [61] estimated the break-even point for a hospital funding of a smoking cessation program for pregnant women and concluded that hospitals would break even at an average cost of $50 per pregnant woman. Break-even costs varied mainly with the incidence of preterm LBW, ranging from $39 to $101 per pregnant woman.

#### 4.3.5. Self-help therapy

We reviewed three studies on self-help as a method for smoking cessation. Lennox *et al.* [62] evaluated the effectiveness and cost-effectiveness of mass-mailing letters encouraging smoking cessation to smokers in a large general medical practice in Scotland, UK. The additional quitters were gained at a cost of between $64 and $153 each, and each additional year of life gained cost between $86 and $210.

Mudde, De Vries, and Strecher [63] estimated the cost-effectiveness of self-help and group counseling in the Netherlands. The study concluded that the self-help method of cessation was at least 3.7 times as cost-effective as the group program. However, the authors pointed out that smokers opting for the self-help program were less addicted and had higher perceptions of self-efficacy than the group in counseling. The total costs to implement the program were $87,176. Total social costs per participant were $211.60. Both methods were considered cost-effective when compared with other medical programs. Brandon *et al.* [64] assessed the effectiveness and cost-effectiveness of a series of booklets *(Stay Quit)* designed to prevent smoking relapse that were mailed to former smokers. They employed a 2x2 randomized factorial design, showing frequency of contact by mail (one mailing vs. eight mailings) and the amount of content in the mailings (one booklet vs. eight booklets). The design cells were therefore (1) low-contact low-content (one booklet), (2) low-contact high-content (all eight booklets were mailed at once), (3) high-contact low-content (repeated letters, where participants were sent one booklet followed by seven short supportive letters over the course of one year), and (4) high-contact high-content (eight separate booklets were sent in eight separate mailings over the course of one year). Follow-up questionnaires that assessed current smoking status were mailed to participants at 12, 18, and 24 months. Results from this study showed that the high-content/high-contact mailings to prevent smoking relapse were cost-effective at reducing relapse rates at $188 per QALY. Results from this study suggested that investment in high-intensity relapse prevention interventions might produce greater benefits than low-intensity interventions.

### 4.4. Other Review Papers on the Economic Evaluations of Cessation

A Cohen and Fowler review of several studies on the economics of smoking cessation up to 1992 [65] was a precursor to a study by Warner [66]. The authors concluded that only a narrow range of available interventions had been subject to economic evaluation and that there was a need for more economic appraisal in this area and for greater consistency in the methodologies used. Furthermore, growing evidence on the effectiveness of pharmacotherapies had not been matched by evidence of their cost-effectiveness, and studies in this area were urgently needed. They also found measures targeted at specific groups were more cost-effective than those targeted at the general population of smokers, and the cost-effectiveness of such targeted programs was further improved by providing educational materials specifically applicable to the targeted groups. Elixhauser [67] reviewed cessation studies conducted in the 1980s and earlier and concluded that there was some evidence that smoking cessation programs tailored to the target population and consisting of multiple interventions with reinforcement of the cessation techniques or messages were cost-effective. Friend and Levy [68] found that the efficacy of brief interventions and cessation treatments in clinical practice had been well studied. However, the impact of population-based interventions on cessation was less well understood. They also concluded the public and private health policies designed to increase access to cessation treatments and information dissemination through brief interventions by health care providers have the potential to increase cessation. Warner [66] summarized the literature on the cost-effectiveness of cessation methods and concluded that low-intensity, and hence low-cost, cessation methods (e.g., self-help materials, brief counseling by non-physician health professionals) saved life years at less cost than more resource-intensive interventions such as NRT. However, this conclusion cannot be generalized because of a lack of consistency of cost and effectiveness across studies. Different cessation methods might have different levels of effectiveness for different groups of people.

In addition to reviewing the economic evaluations of individual tobacco control programs, and summarizing four other review papers on tobacco control programs, we also reviewed a report from the Commonwealth Department of Health and Aging in Australia [69]. This epidemiological and economic evaluation of different public health programs included comprehensive programs to reduce tobacco use over a period of 30 years. The Australian government has undertaken several public health programs such as media campaigns, health warnings, and restrictions in tobacco promotions to reduce consumption. The report showed that reductions in tobacco consumption translated into huge health benefits and concluded that in 1998 an estimated 17,421 premature deaths from coronary heart disease (CHD), lung cancer, chronic obstructive pulmonary disease (COPD) and bronchitis, stroke, and other cancers had been averted. The total value of years of life saved and improved quality of life in 1998 was $12.4 billion (2005 US Dollars). The study also projected an estimated 26,310 deaths from lung cancer, COPD, and CHD will be averted by year 2010 as a result of Australia’s comprehensive programs to reduce tobacco use. Benefits of reduced tobacco consumption were enormous relative to the programs’ costs. A comprehensive economic evaluation of the programs showed a benefit-cost ratio of 50:1. In terms of public finances, every dollar spent on tobacco control programs saved two dollars in government expenses.

## 5. Discussion

Articles reviewed in this paper focused on the effects of tax and pricing policy, government regulations, education, media campaigns, and cessation therapies on smoking prevalence and health outcomes. Smoking cessation therapies and group or individual counseling seem to be the most extensively studied tobacco control interventions. Interventions that combine therapies with some form of counseling targeted at pregnant women are more cost-effective than those with a single intervention. Cessation intervention for pregnant women costs as little as $37 per participant and results in a 15% cessation rate [59]. Therefore, small investments in cessation programs for pregnant women have a favorable impact on pregnancy outcomes. Interventions targeted at pregnant women are successful in reducing the smoking prevalence in this group and in improving LBW and high infant mortality. Ershoff *et al*. [57] study found on average, infants born to experimental group participants were 57 grams heavier, and the incidence of LBW was nearly halved in the experimental group as compared with the control group. The average cost per delivery was $1,767 for the experimental group as compared with $1,846 for the control group. However, Cummings *et al.* [53] and Weiss *et al.* [70] concluded that interventions targeting men were more cost-effective than those for women in terms of the number of quitters and life years gained.

Governments, as well as non-governmental organizations, sponsor various tobacco control programs to alleviate the burden of smoking, and these programs have been effective in reducing smoking initiation, cessation, and exposure to secondhand smoke. Tauras *et al.* [71] examined the relationship between spending on tobacco control and youth smoking. They found that spending on tobacco control significantly reduced youth smoking prevalence and the average quantity of cigarettes smoked.

The demand for cigarettes is affected by several factors including changes in the retail price. Tax-induced price increases have proved to be effective tools in reducing smoking prevalence, which translates to improved health in the long run. A 10% tax-induced increase in cigarette prices, for example, could prevent 5 to 16 million tobacco-attributable deaths worldwide [17]. NRTs and non-price related interventions such as smoke-free-air laws, media campaigns, counseling, and restrictions in cigarette advertising are also effective interventions in terms of reducing prevalence, saving lives, and lowering medical expenditures. The literature also shows that self-help and counseling programs are cost-effective, and the efficiency of such programs is improved by inclusion of NRTs. Benefits from such smoking cessation programs exceed costs and may serve as an incentive for policy makers to endorse other programs such as tax increases on cigarettes and other tobacco products.

Tobacco control interventions are more cost-effective than several other public health interventions. Stone *et al.* [72] came to a similar conclusion, showing that the median cost for tobacco control interventions was $4,400/QALY whereas the median cost for cardiovascular risk counseling was $74,000/QALY and was $18,500/QALY for cancer screening. Maciosek *et al.* [73] ranked preventive services on the basis of their cost-effectiveness scores (1–5 scores) and found that tobacco use counseling, childhood immunization, and aspirin use to prevent cardiovascular events ranked as the most cost-effective preventive services. Other preventive interventions such as cholesterol screening (score: 2), cervical cancer screening (score: 3), breast cancer screening (score: 2), chlamydia screening (score: 4) were less effective than smoking cessation interventions (score: 5). Although the evidence is clear that tobacco use interventions are highly cost-effective, more work needs to be done to rigorously evaluate price and tax increases, media campaigns, smoke free air laws and workplace place interventions, quitlines, youth access enforcement, school and community-based programs.
